# Tune the channel: TRPM8 targeting in prostate cancer

**DOI:** 10.18632/oncoscience.543

**Published:** 2021-09-10

**Authors:** Alessandro Alaimo, Dario De Felice, Sacha Genovesi, Marco Lorenzoni, Andrea Lunardi

**Affiliations:** ^1^Department of Cellular, Computational and Integrative Biology (CIBIO), University of Trento, Trento 38123, Italy

**Keywords:** prostate cancer, TRPM8, precision oncology, cancer translational research

## Abstract

The therapeutic landscape of cancer treatments is quickly evolving thanks to the advent of precision oncology. Discovery of novel druggable targets and more reliable biomarkers is a primary objective towards personalized strategies of cancer treatment. Highly expressed in the prostate epithelium within the human body, Transient Receptor Potential subfamily M member 8 (TRPM8) levels rise in primary and hormone naïve metastatic prostate cancer (PCa) lesions, which makes this channel an interesting prototype of molecular target. Recently, by combining a multidisciplinary approach to an *in vitro* genetic platform, we demonstrated that the combination of potent TRPM8 agonists with X-rays induces a massive apoptotic response in radioresistant pre-malignant and malignant models of primary prostate lesions. As well, TRPM8 activation enhances the efficacy of docetaxel or enzalutamide in eradicating hormone naïve metastatic PCa cells. Overall, our findings provide a solid rationale for pursuing the pre-clinical and clinical study of TRPM8 as a valuable target for future approaches of precise oncology in PCa.

## INTRODUCTION

Prostate Cancer (PCa) is the most commonly diagnosed non-cutaneous cancer in men in the Occident [1]. Despite significant progresses in diagnosis and treatment, PCa remains a primary cause of cancer-related death in both North America and Europe [2]. Patients diagnosed with non-metastatic PCa are highly treatable through surgery or radiotherapy [3]. Contrarywise, patients with high-risk/locally advanced PCa (stage III/IV) account for approximatively 15% of all new cases and have a high probability of tumor recurrence following treatment and dying from the disease [4]. For these subjects - depending on physical and clinical parameters such as life expectancy, patient fitness, Gleason score and PSA levels - the therapeutic protocol foresees radical prostatectomy followed by adjuvant radiotherapy, or radiotherapy coupled with adjuvant systemic chemo/hormonal treatments [4, 5]. Unfortunately, high-risk/locally advanced PCa often progresses to the metastatic stage of the disease and, after few years of androgen deprivation therapy, gives rise to the more aggressive form of PCa known as castration resistant metastatic condition, which is uncurable [6].

In the recent years, the radical expansion of targeted therapies and precision medicine approaches has significantly improved the overall survival of patients affected by different types of liquid and solid tumors. Unfortunately, these strategies are still far from being applicable to PCa [7, 8]. More than ever, the oncology research has the office to identify and characterize molecular targets of clinical relevance. The prevalent expression of molecular targets in cancer cells respect to normal tissues, the druggability, and, finally, the efficacy in killing tumor cells, are all main factors shortening the list of potential valuable candidates. In that regard, ion channels deserve special attention as potential therapeutic targets due to their crucial role in cellular homeostasis and tumorigenesis [9].

Among the growing list of ion channels expressed in normal and tumor prostate epithelial cells, Transient Receptor Potential Melastatin 8 (TRPM8) stands out as one of the most promising clinical targets for PCa treatment [10–12]. Initially identified as a new prostate-specific gene [13], this Ca^2+^-permeable, nonselective cation channel is, along with TRPA1, the primary cold receptor involved in thermosensation in humans. TRPM8 exhibits multiple gating mechanisms, being activated by painless cool or cold temperatures, and chemical ligands such as menthol [10, 14–16]. TRPM8 expression in humans is limited to cold-sensitive sensory neurons and few epithelial tissues, with prostate luminal cells showing the highest levels [13, 17–19]. Interestingly, TRPM8 is overexpressed in primary and hormone naïve metastatic PCa [13, 17, 20–22], which leads to an increased amount of functional channels at the plasma membrane of luminal epithelial cells [21]. On the other hand, the expression levels of this channel is frequently reduced in metastatic castration resistant PCa [13, 17, 20, 22]. Even though the role of TRPM8 in prostate tumorigenesis has been investigated by different groups it remains questioned and controversial [12, 23].

Our *in-silico* analysis of large RNAseq and microarray datasets highlights a substantial inter-tumor heterogeneity of TRPM8 mRNA levels in PCa samples with the full length mRNA generally more abundant compared to its shorter isoforms [22, 24]. Accordingly, immunohistochemistry analysis confirms a heterogeneous distribution of TRPM8 protein amount across human PCa samples with very high TRPM8 staining frequently associated with advanced stage (III/IV) of the disease. Of note, matched primary PCa and hormone naïve metastatic samples derived from the same patient score very similar for TRPM8 immunostaining [22, 24].

Historically, the effectiveness of targeted therapies has often been determined by the expression level of the selected targets in cancer cells (*e.g.,* EGFR, HER2, PDL1) [25, 26]. In line with this very general concept, different channel agonists (Icilin, Menthol, WS-12, D-3263) [27–30] triggered intense calcium currents and massive apoptotic response in immortalized prostate cells (RWPE-1) that were genetically modified to slightly increase the amount of TRPM8 expression. RWPE-1 cells expressing endogenous levels of the channel were completely refractory to the treatment with TRPM8 agonists. To test TRPM8 targeting in genetic models of pre-malignant and malignant prostate lesions, RWPE-1 characterized by endogenous or increased amount of TRPM8 were further manipulated to express, under doxycycline control, ERG alone [31–33], or ERG *plus* PTEN shRNAs (ERG/PTEN-KD) [33, 34]. Severe apoptotic cell death triggered by potent channel agonists in cells expressing increased amount of TRPM8 was completely turned-off in both ERG and ERG/PTEN-KD genetic models, thus suggesting a direct role of ERG in the establishment of a pro-survival cellular program intercepting TRPM8-dependent Ca^2+^ cytotoxicity [22].

New investigational drugs are often tested in two-arm clinical trials to evaluate their efficacy in improving standard-of-care treatments. Taking advantage of our genetic platform, both pre-malignant (ERG) and malignant (ERG/PTEN-KD) models of primary organ confined lesions characterized by endogenous or overexpressed amount of TRPM8 were enrolled in a pre-clinical trial testing the efficacy of combining the potent channel agonist WS-12 with X-rays. Noteworthy, combination of WS-12 with a sub-lethal dose of X-rays overcame single treatments resistance and induced a diffuse apoptotic response in both ERG and ERG/PTEN-KD models characterized by slight TRPM8 overexpression [22].

As previously discussed, the clinical protocol for high-risk/locally advanced PCa patients contemplates adjuvant chemo/hormonal treatments following radiotherapy for primary tumors [35]. Adjuvant therapies aim at eradicating residual disease, which may consist in either local tissues invasion by proximity or in micrometastatic lesions spread in the body, commonly in lymph nodes. The hormone sensitive lymph node derived metastatic prostate cancer cell line LNCaP_FGC_ were choose as the best proxy to study the possible impact of TRPM8 agonists on either enzalutamide or docetaxel efficacy. LNCaP_FGC_ cells were minimally affected by WS-12, docetaxel or enzalutamide when used as single agents. In contrast, combination of WS-12 with either docetaxel or enzalutamide triggers a mild apoptotic cell death response in LNCaP_FGC_ cells expressing endogenous levels of TRPM8 which significantly rise to more than 50% of cells in genetically modified LNCaP_FGC_ slightly overexpressing the channel [22].

In conclusion, the greater expression of TRPM8 in primary and hormone naïve metastatic PCa, the large number of specific agonists [30], recent advances in the use of nanocarriers [36–38] and small molecules to deliver pharmacological compounds [39, 40], and, finally, the possibility to identify PCa patients who could benefit from the administration of TRPM8 agonists in combination with standard-of-care treatments, makes TRPM8 an extremely attractive target for developing more effective clinical protocols based on personalized PCa treatments.
